# Bleeding risk of transbronchial cryobiopsy compared to transbronchial forceps biopsy in interstitial lung disease – a prospective, randomized, multicentre cross-over trial

**DOI:** 10.1186/s12931-019-1091-1

**Published:** 2019-07-05

**Authors:** Juergen Hetzel, Ralf Eberhardt, Christoph Petermann, Wolfgang Gesierich, Kaid Darwiche, Lars Hagmeyer, Rainer Muche, Michael Kreuter, Richard Lewis, Ahmed Ehab, Michael Boeckeler, Maik Haentschel

**Affiliations:** 1 Department of Haematology, Oncology, Rheumatology, Immunology and Pulmology, Eberhard Karls University, Otfried-Mueller-Str. 10, 70771 Tübingen, Germany; 2 Department of Pneumology and Critical Care Medicine, Thoraxklinik, University of Heidelberg and Translational Lung Research Center Heidelberg (TLRCH, German Center for Lung Research (DZL), Heidelberg, Germany; 3 Department for Pulmonary Diseases, Asklepios-Klinik Harburg, Hamburg, Germany; 4 Lung Center Munich West, Helios Klinik Munich West, Munich, Germany; 5 Department of Interventional Pneumology, Ruhrlandklinik, University Hospital Essen, University of Duisburg-Essen, Duisburg, Germany; 6 Clinic for Pneumology and Allergology, Center of Sleep Medicine and Respiratory Care, Bethanien Hospital, Solingen, Germany; 7 Institute of Epidemiology and Medical Biometry, Ulm University Ulm, Ulm, Germany; 8 NPARU, University of Worcester, Worcester, UK

**Keywords:** Interstitial lung disease, Cryobiopsy, Forceps biopsy, Bleeding risk, Randomized prospective multicenter trial

## Abstract

**Background:**

Bronchoscopic cryobiopsy is a new method of bronchoscopic tissue sampling in interstitial lung disease. In case of transbronchial biopsies, the resultant tissue samples are of high quality, and the lung parenchyma seen in the samples is adequate for a histological diagnosis in most cases. Bleeding after transbronchial biopsy is the most important procedure- associated complication and may be life threatening. This study addresses the risk of bleeding of transbronchial cryobiopsy.

**Methods:**

In this prospective, randomized, controlled multicentre study 359 patients with interstitial lung disease requiring diagnostic bronchoscopic tissue sampling were included. Both conventional transbronchial forceps biopsy and transbronchial cryobiopsy were undertaken in each patient. The sequence of the procedures was randomized. Bleeding severity was evaluated semi-quantitatively as “no bleeding”, “mild” (suction alone), “moderate” (additional intervention) or “severe” (prolonged monitoring necessary or fatal outcome), for each intervention.

**Results:**

In 359 patients atotal of 1160 cryobiopsies and 1302 forceps biopsies were performed. Bleeding was observed after forceps biopsy in 173 patients (48.2%) and after cryobiopsy in 261 patients (72.7%).

Bleeding was significantly greater in the cryobiopsy group (cryobiopsy/forceps biopsy: no bleeding 27.3%/51.8%; mild 56.5%/44.0%; moderate 15.0%/4.2%; severe 1.2%/0%; *p* < 0.001). The rate of clinically relevant bleeding (moderate or severe) was higher after the cryobiopsy procedures compared to the forceps biopsies (16.2% vs. 4.2%, *p* < 0.05). No fatal bleeding complications occurred.

**Conclusions:**

Compared to transbronchial forceps biopsy, transbronchial cryobiopsy was associated with an increased risk of bleeding which is of clinical relevance. Therefore training and additional precautions for bleeding control should be considered.

**Trial registration:**

The study was registered with clinicaltrials.gov (NCT01894113).

## Background

Diagnosis of interstitial lung diseases remains a challenge. Although, in some cases, the diagnosis can be made on the basis of clinical and radiological data alone, tissue sampling for histological evaluation is needed in a significant proportion of patients [1–3]. However, distinguishing the different types of interstitial lung disease (ILD) is crucial due to its prognostic impact and informing different therapeutic interventions. With a diagnostic yield of 86 to 92% surgical lung biopsy (SLB) exceeds the sensitivity of conventional transbronchial lung forceps biopsy (TBFB) and is therefore recommended in the current guidelines for adequate tissue sampling in patients with suspected idiopathic interstitial pneumonias [4–7]. However SLB’s are associated with a significant mortality rate - 1.7% in elective patients, and up to between 16% [8] and 21.7% [9] in non-elective patients. These data highlight the need for less invasive, and safer, high yield diagnostic sampling methods.

Transbronchial lung cryobiopsy (TBCB) now meets this need with its larger specimens [10, 11] and reduced tissue artefacts [10, 12, 13], markedly improving the diagnostic yield compared to TBFB [10, 14–17]. In combination with clinical and radiological information, TBCB provides substantial additional diagnostic information and reaches a similar confidence level in idiopathic pulmonary fibrosis (IPF) diagnosis to that of SLB [18]. However it is unclear what the cost of this additional information is in terms of a higher complication rate. Several, mostly retrospective papers, reported high pneumothorax rates, severe bleeding complications and a few cases of fatal exacerbations after transbronchial cryobiopsies [19, 20]. Whereas the number of exacerbations was low, and pneumothoraces could be managed relatively easily, bleeding after TBCB remains the main concern.

To establish comparative safety profiles we performed a trial to evaluate complication rates of TBCB compared to TBFB in patients with ILD requiring tissue sampling for diagnosis.

## Methods

This prospective, randomized, multicentre study was performed in six pulmonary centers in Germany.

### Study population

Patients with radiologically proven ILD requiring histological examination for further evaluation were chosen for inclusion in this study. All patients were over 18 years old. Signed informed consent was mandatory. Exclusion criteria included any possible bleeding disorders (international normalized ratio (INR) > 1.3, partial thromboplastin time (PTT) above normal range, thrombocytopenia < 100.000/μl), treatment with thienopyridines, oxygen saturation below 90% after delivery of oxygen at a maximum flow rate of 2 l per minute, pre-existing severe cardiac diseases (e.g. instable angina pectoris, myocardial infarction, decompensated cardiac insufficiency) or known echocardiographically measured pulmonary hypertension with a systolic pulmonary artery pressure greater than 50 mmHg. While treatment with clopidogrel and other new antiplatelet drugs had to be stopped periinterventionally, treatment with acetylsalicylic acid could be continued.

### *Bronchoscopy*

Bronchoscopy was performed with either a flexible or rigid technique, depending on the operator’s choice. In case of flexible bronchoscopy placement of an endotracheal tube was recommended in order to provide a secure airway. When rigid bronchoscopy was undertaken, tissue biopsy was carried out by using the flexible bronchoscope inserted through the rigid tube. General anaesthesia for rigid bronchoscopy, deep sedation and local anaesthesia for intubation with a flexible tube and patients’ monitoring, including continuous oxygen saturation, ECG monitoring and repeated non-invasive blood pressure monitoring, were performed according to the local standard of each centre.

### Tissue sampling

In every patient both conventional transbronchial forceps and cryobiopsy techniques were performed. The sequence of the biopsy techniques was randomized. Fluoroscopy guidance was recommended for both techniques. For cryobiopsy a distance of 1 cm from the visceral pleura was recommended. TBCB was performed using cryoprobes of 1.9 or 2.4 mm diameter (Erbe Elektromedizin GmbH, Tübingen, Germany) and a freezing time of 3 to 7 s depending on the freezing power and diameter of the cryoprobe. TBFB biopsy was performed using forceps of a diameter between 1.8 mm and 2.6 mm.

### Data acquisition

Demographic data were documented. Bleeding was predefined as described below.

### Evaluation of bleeding

The different biopsy techniques TBCB and TBFB were evaluated for occurrence of bleeding. As several biopsies were taken using each technique, the most severe bleeding from each technique was documented. Bleeding severity was determined on a four-point scale: *none*; *mild bleeding*: (self-limiting bleeding, manageable with suction alone and without the need for any specific intervention); *moderate bleeding*: (use of any additional intervention such as instillation of ice-cold saline or vasoconstrictive drugs, or transient balloon tamponade in order to prevent bleeding in the central airways); *severe bleeding*: additional prolonged monitoring or intensive care therapy after the procedure was necessary or if the bleeding was fatal. Prophylactic balloon placement was not performed.

### Evaluation of pneumothorax

Occurrence of pneumothorax was evaluated by conventional chest X-ray between one and two hours following the bronchoscopy.

### Randomization

The sequence of biopsy techniques (TBFB or TBCB) was randomized using consecutive numbered envelopes for randomization at each study site. The Institute of Epidemiology and Medical Biometry, Ulm University, Ulm, Germany, provided randomization envelopes and reviewed adherence to randomization at the end of the study.

### Statistics

The incidence and severity of bleeding, being the primary aim of this study, was given by relative and absolute frequencies and were compared using the McNemar-Bowkertest for categorical and Wilcoxon signed rank test for continuous outcomes. Carry over effect was ruled out beforehand. Fisher’s exact test was used for comparisons in contingency tables for independent (sub)groups. A p-level < 0.05 was regarded as significant. Statistical analysis was done by JMP 13.1 and supported by the Institute of Epidemiology and Medical Biometry, Ulm University, Germany.

The study design and protocol was approved by the Ethics Committees of Tuebingen (Reference number 035/2011MPG23) and confirmed by each individual site ethics committee*.*

## Results

**Fig. 1:**
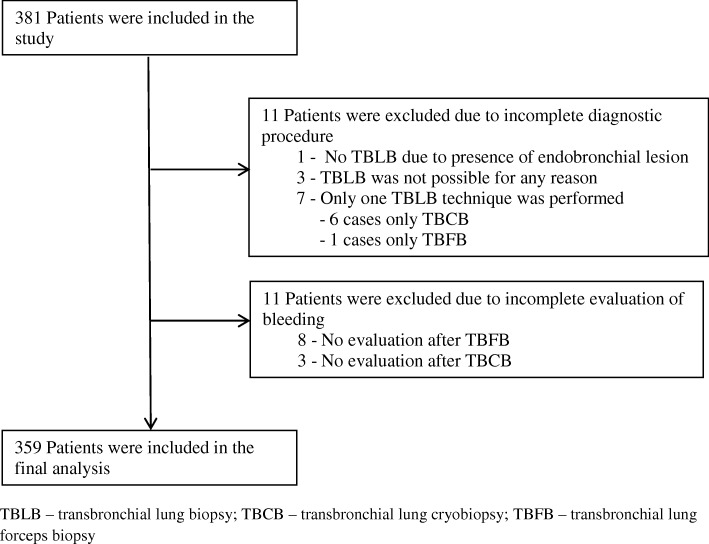
Study population. TBLB – transbronchial lung biopsy; TBCB – transbronchial lung cryobiopsy; TBFB – transbronchial lung forceps biopsy

In this multicentre study a total number of 381 patients were included in 6 German pulmonary centres. 11 randomized patients had to be excluded due to an incomplete diagnostic procedure with none, or only one, biopsy technique (Fig. 1). In 6 patients the second biopsy technique was not performed because of moderate bleeding after the first technique (in 5 patients after TBCB, in one case after TBFB). In one case a pneumothorax after TBCB occurred, thus TBFB was not performed. In the remaining 370 patients bleeding quantification was missing in 8 patients in the forceps biopsy group and in 3 patients in the cryobiopsy group, resulting in a final study population of 359 patients.

### Patient characteristics

Patient characteristics are summarized in Table 1.

**Table 1:** Characteristics of the Patients*

Variable	*N* = 359 (100%)
Age – yrs	62.8 (±14.0)
Sex – no. (%)	
Female	153 (42.6)
Male	198 (55.2)
Weight – kg	78.0 (±15.7)
Body height – cm	169.3 (±10.0)
Smoking status – no. (%)	
Non-smoker	132 (36.8)
Ex-smoker	138 (38.4)
Current Smoker	62 (17.3)
Smoking index – pack years	
Ex-smoker	24.9 (±18.8)
Current smoker	32.1 (±20.9)
Coagulation profile	
Prothrombin time (INR)	1.02 (±0.12)
Partial thromboplastin time (PTT) – sec	28.8 (±5.8)
Thrombocytes – × 1000/μl	271 (±161)
Aspirin – no. (%)	
Yes	51 (14.2)
No	303 (84.6)

*Plus-minus values are means ± Standard deviation (SD). Patient characteristics were unknown for age in 10 patients (2.8%), for sex in 8 patients (2.2%), weight and height in 17 patients (4.7%), smoking status in 27 patients (7.5%), prothrombin time (INR) in 8 patients (2.2%), partial thromboplastin time (PTT) in 27 patients (7.5%), thrombocyte count in 15 patients (4.2%) and for aspirin intake in 5 patients (1.4%)

### Bronchoscopic intervention

Specification of the bronchoscopic intervention, number of biopsies which each technique, sedation, intubation technique and size of biopsy probes are summarized in Table 2. Localization for biopsy site, and the size of the biopsy tool was left to individual choice. TBCB was performed in just one lobe in 205 patients, and from 2 different lobes in 151 patients, with a total of 1160 cryobiopsies being taken. Forceps biopsy was performed in one lobe in 166 patients and multiple lobes in 189 patients. Forceps biopsies were performed in multiple lobes in significantly more patients (*p* = 0.004), with a total of 1302 biopsies. Forceps biopsies were taken in the upper lobe in 39.6% and in the lower lobe in 60.4% of patients, whereas cryobiopsies were taken in the upper lobe in 36.0% and in the lower lobe in 64.0% of patients. There were significantly more biopsies performed in the right lung compared to the left lung (cryobiopsy: 812 (70.0%), forceps biopsy: 900 (69.1%)) (Table 2).

**Table 2:** Bronchoscopic intervention*

Variable	N = 359 (100%)	
Number of biopsies		
Cryobiopsy		
Total number of cryobiopsies	1160	
Cryobiopsies per patient	3,2 ± 1,2	
Forceps biopsy		
Total number of forceps biopsies	1302	
Forceps biopsies per patient	3,6 ± 1,2	
Sedation – no. (%)		
General anaesthesia	169 (47.1)	
Deep sedation	185 (51.5)	
Deep sedation		
Midazolam		
Number of patients	115	
Dose – mg	3.57 (±1.66)	
Propofol		
Number of patients	168	
Dose – mg	315.2 (±166.9)	
Intubation technique – no. (%)		
Rigid bronchoscope	169 (47.6)	
Endotracheal tube intubation	182 (51.3)	
No intubation	4 (1.1)	
Probe size		
Cryobiopsy – no. (%)		
Small		
1.9 mm	212 (59.1)	
Large		
2.4 mm	145 (40.4)	
Forceps – no. (%)		
Small		
total	169 (47.1)	
1.8 mm	145 (40.4)	
2.0 mm	24 (6.7)	
Large		
total	186 (51.8)	
2.2 mm	161 (44.8)	
2.6 mm	25 (7.0)	
	Biopsy technique	
	TBCB	TBFB
Localization of biopsy - no. (%)		
Biopsy in different lobes	151 (42.1)	189 (52.6)
Biopsy in just one lobe	205 (57.1)	166 (46.2)
Right upper lobe and middle lobe	284 (24.5)	357 (27.4)
Left upper lobe	134 (11.6)	159 (12.2)
Right lower lobe	528 (45.5)	543 (41.7)
Left lower lobe	214 (18.4)	243 (18.7)

*Plus-minus values are means ± Standard deviation (SD). Data were unknown as follows: for sedation in 5 patients (1.4%), midazolam application in 15 patients (4.2%), propofol application in 30 patients (8.4%), intubation technique in 4 patients (1.1%), probe size of cryobiopsy in 1 patient (0.3%), and probe size of forceps in 4 patients (1.1%) and localization of cryobiopsy/ forceps biopsy in 4/ 4 patients (1.1/ 1.1%). TBCB – transbronchial lung cryobiopsy; TBFB – transbronchial lung forceps biopsy

### Incidence and severity of biopsy related bleeding

**Fig. 2:**
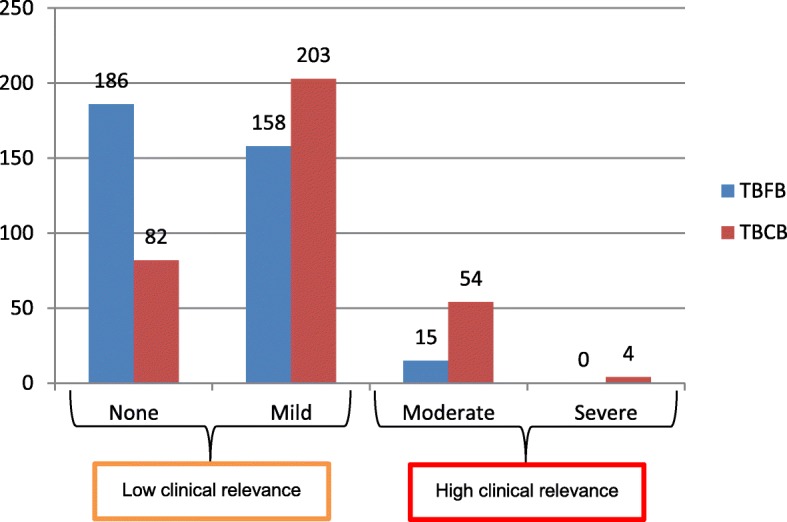
Severity of biopsy-related bleeding, comparing both techniques. (TBCB – transbronchial lung cryobiopsy; TBFB – transbronchial lung forceps biopsy)

The severity of biopsy related bleeding comparing the two different biopsy techniques is summarized in Table 3 and Fig. 2. Bleeding severity was significantly higher after TBCB compared to TBFB. Using the categorization of bleeding described above, we observed none, mild, moderate and severe bleeding in 98 (27.3%), 203 (56.5%), 54 (15.0%) and 3 (1.1%) patients after TBCB compared to 186 (51.8%), 158 (44.0%), 15 (4.2%) and 0 (0%) cases after TBFB (*p* < 0,001). Bleeding occurred more often in the TBCB group (bleeding in 261 cases (72.7%), none in 98 cases (27.3%)) compared to the TBFB group (bleeding in 173 cases (48.2%), none in 186 cases (51.3%)) (*p* < 0.001).

**Table 3:** Severity of biopsy- related bleeding*

TBCB N = 359 (100%)				
Bleeding– no. (%)	None 98 (27.3)	Mild 203 (56.5)	Moderate 54 (15.0)	Severe 4 (1.1)
TBFB N = 359 (100%)				
None 186 (51.8)	82 (22.8)	88 (24.5)	14 (3.9)	2 (0.6)
Mild 158 (44.0)	14 (3.9)	111 (30.9)	31 (8.6)	2 (0.6)
Moderate 15 (4.2)	2 (0.6)	4 (1.1)	9 (2.5)	0 (0)
Severe 0 (0)	0 (0)	0 (0)	0 (0)	0 (0)

Severity of biopsy-related bleeding, comparing both techniques (TBCB – transbronchial lung cryobiopsy; TBFB – transbronchial lung forceps biopsy) for each patient. Severity of bleeding was categorized as no, mild, moderate and severe bleeding as defined above. Difference in bleeding incidence was significant different over all categories (*p < 0.001, calculated by the Bowker’s test)

**Table 4:** Incidence and severity of bleeding related to patient characteristics*

		Technique					
		Cryobiopsy (N = 359)			Forceps biopsy (N = 359)		
Bleeding severity		None/Mild	Moderate/Severe	p-value	None/Mild	Moderate/Severe	p-value
Clinical relevance		Low	High		Low	High	
all – no (%)		301 (83.8)	58 (16.2)	–	344 (95.8)	15 (4.2)	–
	n						
Age	349						
< 65 yr	160	143	17	0.018	155	5	0.59
> = 65 yr	189	151	38		180	9	
Sex	351						
Female	153	121	32	0.026	145	8	0.41
Male	198	175	23		192	6	
Body height	342						
= < 170 cm	186	146	40	0.002	175	11	0.043
> 170 cm	156	142	14		154	2	
Smoking	332						
Non-smoker	132	109	23	0.52	124	8	0.27
Ex-smoker	138	118	20		135	3	
Current smoker	62	55	7		59	3	
Aspirin	354						
Yes	51	38	13	0.067	49	2	1.0
No	303	258	45		290	13	

*Clinical relevance of bleeding is categorized as “low” (none, and mild bleeding) and “high” (moderate and severe bleeding) for both biopsy techniques. Absolute number (n) of evaluable patients are listed in the second column. Data were missing for age in 10 patients (2.8%), sex in 8 patients (2.2%), body height in 17 patients (4.7%), smoking status in 27 patients (7.5%), and for aspirin use in 5 patients (1.4%). *P*-values were calculated by 2 tail Fisher’s exact test for independent samples focusing on each technique separately

Bleeding severity was categorized as bleeding of low clinical relevance (none or mild) and high clinical relevance (moderate or severe). We observed a significantly higher incidence of bleeding of high clinical relevance (moderate or severe) in the TBCB group (58 patients (16.2%)) compared to the TBFB biopsy group (15 patients (4.2%) (*p* < 0,001)). There were no cases of severe bleeding after TBFB, but 4 cases after TBCB. They had a normal coagulation status, one patient was on aspirin. In those 4 patients endobronchial vasoconstrictive drugs were instilled, and in two cases a local bronchial blockage with a Fogarty balloon was also applied. In all cases bleeding could be controlled during the bronchoscopy. One of those patients, who had already suffered from an exacerbation of his fibrosing interstitial lung disease prior to the bronchoscopy, died within 30 days after bronchoscopy. After the bronchoscopy and biopsy with both techniques, oro-tracheal intubation and transient mechanical ventilation was needed because he had respiratory failure. One day after the procedure he could be extubated. However one day after extubation he developed acute cardiac insufficiency with consecutive respiratory failure, which could not be stabilized, and the patient died 6 days after the procedure.

The bleeding severity wasn’t influenced by the biopsy sequence for either technique.

### Incidence/severity of bleeding regarding patient characteristics and bronchoscopic intervention

In the TBCB group we found significant more bleeding of high clinical relevance (moderate/severe) in those age > = 65 years compared to those < 65 years (Table 4). In the TBCB group we observed significantly more highly clinically relevant bleeding in patients with a height < = 170 cm. Furthermore females showed a significant higher clinically relevant bleeding rate than men when the biopsies were taken with the cryoprobe. Similarly in the TBFB we observed significantly more highly clinically relevant bleeding in patients <= 170 cm than those > 170 cm. However there was no relationship comparing bleeding with sex and age. Comparison of bleeding severity independent of the applied biopsy technique (TBCB and TBFB) showed significant more bleeding in older patients (> = 65 yrs), females, and patients with a body height < =170 cm.

There were no significant differences in bleeding of either low or high clinical relevance in relation to coagulation profile (INR, PTT or thrombocyte count) between TBCB and TBFB. Treatment with low dose aspirin showed a trend (*p* = 0.067) to more highly clinically relevant bleeding in the TBCB group, whereas no difference could be observed in the TBFB group.

Analyses of biopsy-related bleeding in relation to different bronchoscopic intervention parameters (biopsy localization and probe size) are shown in Table 5. Use of small probes compared to large probes showed significantly less clinically relevant bleeding in the cryobiopsy group (*p* = 0.006) whereas in the forceps biopsy group a trend to less bleeding with small probes could be observed (*p* = 0.12). Comparison of the small cryobiopsy probe with the large forceps biopsy probe showed significantly more clinically relevant bleeding with the cryobiopsy (*p* < 0.001).

**Table 5:** Incidence and severity of bleeding regarding bronchoscopic intervention

		Technique				
		Cryobiopsy (N = 359)		Forceps biopsy (N = 359)		
Bleeding severity		None/Mild	Moderate/Severe	None/Mild	Moderate/Severe	*p*- value
Clinical relevance		Low	High	Low	High	
Intervention – no (%)		301 (83.8)	58 (16.2)	344 (95.8)	15 (4.2)	TBCB vs TBFB < 0.001
Probe size						
Cryobiopsy	357					
Small: 1.9 mm	211	187	25			SC vs. LC: *p* = 0.006 SC vs. LF: *p* < 0.001
Large: 2.4 mm	146	112	33			
Forceps	355					
Small: 1.8–2.0 mm	169			165	4	SF vs. LF: *p* = 0.12
Large: 2.2–2.6 mm	186			175	11	
Localization of biopsy						
Upper lobe		48	13			*p* = 1.0
Lower lobe		112	32			
Upper lobe				49	4	*p* = 0.27
Lower lobe				109	4	

*Clinical relevance of bleeding is categorized as “low” (None, and mild bleeding) and “high” (moderate and severe bleeding) for both biopsy techniques. Absolute number of evaluable patients is listed in the second column. P-values were calculated by 2 tail Fisher’s exact test for independent samples, comparing small cryobiopsy (SC) and large cryobiopsy (LC), small forceps biopsy (SF) and large forceps biopsy (LF) as well as small cryobiopsy (SC) and large forceps biopsy (LF)

In case of bleeding of moderate to severe categories, a combination of one or more of the following bleeding control interventions was performed: instillation of drugs (cold saline in 49 cases, Xylometazolin in 62 cases, adrenalin in 11 cases, tranexamic acid in 1 cases), balloon tamponade in 13 cases, temporary tamponade a gauze compress in 3 cases and wedging with the bronchoscope followed by intubation and controlled ventilation in one case.

### Correlation between relevance of bleeding and patient’s height

Since both the size of cryoprobes and the patient’s’ height were associated with bleeding severity, we analysed relevance of bleeding (as calculated by the ratio of relevant bleeding to non-relevant bleeding) in relation to both factors. The increase of clinically relevant bleeding with the large cryoprobe in comparison to the small cryoprobe was higher in patients <= 170 cm (45.3 vs 17.2%, 2.63fold) compared to patients > 170 cm (12.3 vs 8.3%, 1.47fold) (Table 6). Whereas the proportion of relevant bleeding was significantly higher with the large cryoprobes in patients = < 170 cm (*p* < 0.05), this was not the case for patients > 170 cm.

**Table 6:** Influence of the size of the cryoprobe and patient’s height on clinical relevance of bleeding

Relevant bleeding/Non- rel. Bleeding (%)		Patient‘s height	
		= < 170 cm	> 170 cm
Size of cryo probe	Small (1.9 mm)	17.2	8.3
	Large (2.4 mm)	45.3	12.3

### Incidence of periinterventional pneumothorax

27 patients (7.5%) developed a pneumothorax after TBCB and TBFB. There was no significant difference in the rate of pneumothoraces between patients having TBCB with the small or the large cryoprobes.

## Discussion

Bleeding is the main peri-procedural complication of interventional bronchoscopic techniques and is also the main complication of TBCB. This may be a life-threatening situation and is therefore of high clinical relevance. The severity of bleeding is very difficult to quantify as it is influenced by two factors which are difficult to measure. The first factor is the bleeding quantity - the absolute amount of endobronchial blood. The second, probably more important factor, is the bleeding intensity – the blood flow per unit time. The volumes in each case may also be influenced by additional fluids inserted via the bronchoscope, and the variable retrieval rate of any intra-bronchial fluid. Therefore current rating classifications of bleeding severity are based on subjective assessments. Studies evaluating TBCB have reported a wide range of bleeding complications, which illustrates the lack of a valid scale [19].

In order to overcome this limitation we have combined three approaches to quantify bleeding.

Firstly we have used the clinical and therapeutic consequences of endobronchial bleeding as criteria to scale bleeding severity semi-quantitatively with “bleeding was controlled by suction alone” as “mild” bleeding or “the patient had to have prolonged monitoring or intensive care therapy due to the bleeding” as “severe” bleeding. This method based on clinical consequences exactly mirrors the clinical relevance.

Secondly we compared bleeding caused by TBCB as a “new” technique with a well-known and established technique. We chose the TBFB as the comparator to TBCB. This strategy offers two advantages: firstly, the severity of bleeding after TBFB is well known among bronchoscopists, and therefore bleeding after TBCB can be assessed in relation to TBFB much more easily and precisely. The second advantage is that the use of a comparator minimizes individual subjective variance in the rating of the two techniques, although the use of any scale based on clinical and bronchoscopic consequences remains subjective and thus variable between individuals. However, since each bronchoscopist applies their own subjective rating scale on both biopsy techniques, comparison is valid.

Thirdly, we compared bleeding severity of both biopsy techniques in the same patient in a randomized sequence in order to minimize the influence of individual patient characteristics such as the underlying lung disease, coagulation status or pre-procedure haemodynamic status.

This prospective randomized multicentre trial has revealed that TBCB caused a higher incidence and significantly more clinically relevant endobronchial bleeding compared to TBFB. In each participating centre a trend towards more bleeding in the cryobiopsy group could be observed. Any bias within a single centre is reduced by the multi-centre design of the study. In the cryobiopsy group 84% of the patients showed no bleeding at all, or only a mild bleeding controllable by suction alone, compared to 96% in the forceps group. Thus in 16% of the patients after TBCB additional procedures had to be performed in order to control bleeding, confirming greater clinically relevant bleeding after TBCB than TBFB. It has to be emphasized that a prophylactic balloon placement for protection of the central airways, as described in other studies [21], was not been performed in this study. Therefore the judgement on bleeding severity in the cryobiopsy group wasn’t compromised by the tamponade, and therefore true comparison of bleeding between the two groups has been possible.

There was a great diversity of methods used to control bleeding in this study. This variety in technique for bleeding control indicates that there is currently no consensus as to the best method for bleeding control, and this area requires further study. Comparing the principal methods, endobronchial installation of drugs and endobronchial tamponade, it is clear that only tamponade results in the immediate protection of the non-biopsied lung areas.

The number of biopsies taken could have influenced the results; the more biopsies, the higher the probability of more severe bleeding. The median number of biopsies performed was higher for TBFB (3.6 biopsies per patient) compared to TBCB (3.2 biopsies per patient). Therefore, the observed difference in bleeding severity cannot be explained by the difference in the number of biopsies of the two different techniques. The sequence of the biopsy techniques did not influence bleeding.

The bleeding severity could also have been influenced by the site of each biopsy. Although fluoroscopy was used to determine comparable biopsy areas, equality of the distance to the pleura cannot be guaranteed by either fluoroscopy or by withdrawal distance from the position where the probe cannot be passed further. Bleeding may have also been influenced by any imbalance in the number of biopsies from different lobes of the lung. We observed a tendency towards a higher proportion of TBFB (40%) in the upper lobe compared to TBCB (36%, *p* = 0.066), which may have influenced our results. To exclude this potential bias we compared bleeding severity in those patients in whom a certain technique was performed only in one lobe, but not in different lobes. We couldn’t observe any difference in bleeding severity between the lower lobe and the upper lobe for each technique. Therefore it is unlikely, that the tendency towards an imbalance in the distribution of biopsies between upper and lower lobe has influenced our results. In a similar manner, the difference in the higher proportion of patients in the forceps group, who were biopsied in both lobes, does not appear to have affected the bleeding rate either.

Interestingly, body height correlated with a higher bleeding risk: the shorter the patient the higher the bleeding risk. In addition in the cryobiopsy group female patients showed a significantly higher bleeding risk than male patients. These differences are difficult to explain as multiple factors may have influenced these results. However, one explanation has to be considered, namely that body height is correlated with the size of the lungs. Therefore the relative size of the biopsy tool in relation to the size of the bronchi is increased in smaller patients. As a result, positioning of the cryoprobe in the small bronchioli of the lung periphery, where the blood vessels are smaller, may be more difficult to achieve in smaller patients. Furthermore in relation to the pulmonary structures the resultant biopsy samples are bigger. Both factors may increase the risk of damaging larger vessels, and therefore increase the risk of bleeding. This hypothesis is supported by the fact that the relative increase of bleeding-risk with a large cryoprobe compared to a small cryoprobe is higher in shorter patients compared to taller patients (large cryoprobe: 2.63 fold increase; small cryoprobe 1.47 fold increase).

However, one limitation of the subgroup analyses is that we assumed a relation between body height and lung volume, which is quite reasonable, but this was not measured in the study setting and therefore confirmatory data is not available. Gender and age also influence the lung size with female and older patients having smaller lungs. As female patients and older patients showed a higher bleeding risk in the cryobiopsy group this correlation supports the causal relationship between lung size and bleeding risk.

As treatment with aspirin has not been shown to be associated definitively with an increased risk of bleeding, patients on aspirin were included in the present trial [22]. Our present data confirm these previous results, with a bleeding risk after forceps biopsy which was not different with or without aspirin treatment. However the low incidence of clinically relevant bleeding limits the validity of such statistical testing.

In contrast to forceps biopsy, in the cryobiopsy group there was a tendency to more severe bleeding in patients treated with aspirin. In combination with the higher absolute number of moderate and severe bleeding, this may be clinically relevant.

Another complication of TBCB is the rate of pneumothoraces. Although it is impossible to assign any pneumothorax to either forceps or cryobiopsies in this study setting, the overall rate of 7.5% was relatively low in comparison to other studies [19]. It has to be emphasized that the number of cryobiopsies per patient was comparable to the other studies. Therefore the lower amount of pneumothoraces compared to previous cryobiopsy studies cannot be explained by a lower number of cryobiopsies. It is possible that the low number of pneumothoraces in our study may be due to operator’s experience, since most published studies relate to an earlier period following development of the technique.

We observed one fatal outcome in a patient with acute exacerbation prior to the intervention; this underlines the importance for strict selection of stable patients for transbronchial biopsy.

In summary, this prospective, randomized, multicentre and so far the largest study on TBCB, reveals that bleeding is a clinically relevant risk factor in TBCB which is greater than for TBFB. Based on this observation, with a frequency of 16% for clinically relevant bleeding after TBCB, it can be concluded that any procedure using TBCB should ensure protection of the airways, either by using rigid bronchoscopy or the placement of an endotracheal tube. Also precautions for bleeding control, such as prophylactic balloon placement, are recommended. Methods to increase the safety of TBCB increase its risk/benefit ratio and therefore justify the use of TBCB to obtain the superior histological and clinically relevant material. It is necessary to place the risk of TBCB in the context with the risk of a more invasive surgical biopsy when making decisions as to the most relevant biopsy procedure used to obtain diagnostic histological material in each individual patient. This study has not directly compared the two methods of TBCB vs surgical biopsy, and such comparisons are difficult with the large numbers of possible variables including patient selection. However, our study has clearly shown that TBCB can be carried out without significant complication in the majority of patients.

## Conclusion

Our study shows that transbronchial cryobiopsy was associated with an increased risk of bleeding compared to transbronchial forceps biopsy. This side effect is clinically relevant. Therefore training and additional precautions for bleeding control should be considered.

## Data Availability

The datasets used and/or analyzed during the current study are available from the corresponding author on reasonable request. For any further analysis ethics vote and patients consent is mandatory.

## Abbreviations

ILD: Interstitial lung disease
INR: International normalized ratio
IPF: Idiopathic pulmonary fibrosis
PTT: Partial thromboplastin time
SLB: Surgical lung biopsy
TBCB: Transbronchial cryobiopsy
TBFB: Transbronchial forceps biopsy
TBLB: Transbronchial lung biopsy
